# Obesity and the Incidence of Bladder Injury and Urinary Retention Following Tension-Free Vaginal Tape Procedure: Retrospective Cohort Study

**DOI:** 10.1155/2011/746393

**Published:** 2011-06-22

**Authors:** Vladimir Revicky, Sambit Mukhopadhyay, Frances de Boer, Edward P. Morris

**Affiliations:** Department of Obstetrics and Gynaecology, Norfolk and Norwich University Hospital, Colney Lane, Norwich NR4 7UY, UK

## Abstract

*Background/Aims*. Aim of the study was to establish an effect of obesity on the incidence of bladder injury or urinary retention following tension-free vaginal tape (TVT) procedure. *Methods*. This was a retrospective cohort study based at the Norfolk and Norwich University Hospital in the UK. Study population included 342 cases of TVT procedures. Incidence of bladder injury was 4.7% (16/342). Rate of urinary retention was 9% (31/342). Body mass index (BMI), age, type of analgesia, concomitant prolapse repair, and previous surgery were factors studied. Univariate analysis was performed to establish a relationship between BMI and complications, followed by a multivariable regression analysis to adjust for age, concomitant surgery, type of analgesia, and previous surgery. *Results*. Neither univariate analysis nor multivariate regression analysis revealed any statistically significant influence of obesity on the incidence of bladder injury or urinary retention. Unadjusted odds ratios and adjusted odds ratios for bladder injury and urinary retention by BMI groups were OR 1.7296 CI 0.4818–6.2097; OR 1.3745 CI 0.5718–3.3043 and adj. OR 2.885 CI 0.603–13.8; adj. OR 1.299 CI 0.502–3.365. *Conclusion*. Obesity does not appear to influence the rate of bladder injury or urinary retention following TVT procedure.

## 1. Introduction

An involuntary urine leakage on effort or exertion or on sneezing or coughing without increase in detrusor pressure is defined as stress urinary incontinence (SUI) [1]. The prevalence of SUI in nulliparous women is estimated to 4.7%, age-standardized prevalence in women with history of Caesarean section is 6.9% and with history of vaginal delivery is 12.2% [2]. However, some reports estimated that SUI may be affecting up to 30% of women [3]. Pathophysiology of SUI was explained by hypermobility of the urethra and bladder neck during exertion [4]. Provided, surgical treatment for SUI is considered, retropubic mid-urethral tape procedure using a “bottom-up” approach with macroporous polypropylene meshes is recommended [5]. In 1996, tension-free vaginal tape (TVT) procedure was described for the first time and it quickly became one of the most popular procedures worldwide [6, 7]. Although TVT is considered minimally invasive, it carries risk of immediate surgical complications as bladder injury and injury to pelvic vessels and bowel [8–10]. Further, urge incontinence and voiding dysfunction are recognised postoperative complications [8, 10]. Currently, there is conflicting evidence on the effect of obesity on peri- and postoperative complications in previous studies. Obesity is defined by a body mass index (BMI) that is equal or greater than 30 kg/m^2^. In 2003, estimated prevalence of overweight and obese women was 56% [11]. As a result, the aim of this study was to determine the effect of obesity on the incidence of bladder injury and urinary retention following tension-free vaginal tape procedure. 

## 2. Materials and Methods

This was a retrospective cohort study based at the Norfolk and Norwich University Hospital in the UK. TVT was defined as retropubic mid-urethral tape procedure using a “bottom-up” approach with macroporous polypropylene meshes. All procedures were performed in the urogynaecological unit. Data for the statistical analysis was obtained from the urogynaecological unit database collecting all cases of TVT. The primary outcome was occurrence of bladder injury and urinary retention. Bladder injury was defined as injury caused by trocars for introduction of TVT confirmed with perioperative cystoscopy. Urinary retention was defined as an inability to pass urine of 200 mL within 6 hours of TVT procedure or postvoiding volume of more than 150 mL. BMI (kg/m^2^), age, type of analgesia, concomitant prolapse repair, and previous surgery were studied factors. General anaesthesia, spinal anaesthesia, and sedation with local anaesthesia were used for TVT procedures. After assuring that a distribution of BMI across categories of complications was equal, study cohort was stratified to two subgroups by BMI (<30 and ≥30). Medians and interquartile ranges were used to describe continuous variables and the Mann-Whitney *U* test and the Kruskal-Wallis test were used for comparisons of continuous data. Chi-square test was used for univariate analysis of categorical data to compare subgroups and to establish a relationship between BMI and bladder injury or urinary retention. This was followed by a multivariable regression analysis to adjust this relationship for age, concomitant surgery, type of analgesia, and previous surgery. Unadjusted and adjusted odds ratios with 95% confidence intervals (CI) were calculated. 

Statistical analysis was performed using SPSS computer software (statistical package for the social sciences, version 16.0).

## 3. Results

Study population of 342 cases of TVT procedures were analysed during the period of 2001–2008. The incidence of bladder injury was 4.7% (16/342) and the rate of urinary retention was 9% (31/342). Cohort was stratified into two subgroups by body mass index (<30, ≥30) with following description: *n* = 246 (72%) and 96 (28%), respectively, and median 25.5 with an interquartile range (23–27) and 33 (31–35), respectively. There was no statistically significant difference in the cohort characteristics by BMI subgroups (age, concomitant procedure, type of analgesia, previous surgery, and complications) (Table 1). The distribution of BMI was the same across categories of complications (the Kruskal-Wallis test, *P* = 0.675). Neither univariate analysis nor multivariate regression analysis revealed any statistically significant influence of BMI (<30, ≥30) on the incidence of bladder injury or urinary retention (Table 2). Unadjusted odds ratios (OR) and adjusted odds ratios (adj. OR) for bladder injury and urinary retention by BMI groups were OR 1.7296 CI 0.4818–6.2097; OR 1.3745 CI 0.5718–3.3043 and adj. OR 2.885 CI 0.603–13.8; adj. OR 1.299 CI 0.502–3.365. Posthoc statistical power analysis was performed using McFadden R-Squared 0.085, with statistical power 0.996.

## 4. Discussion

In 2003, Department of Health estimated prevalence of overweight and obese women to 56%. Further 26% raise in obesity occurred in 2010 [11]. This epidemic increase will put strains on urogynaecological services. In this light, it is important to predict the impact of BMI on procedures such as TVT. Several studies produced conflicting findings of the effect of BMI on perioperative and postoperative complications. 

Two studies reported on increased incidence of a bladder injury with BMI less than 30 kg/m^2^ [12, 13]. Nevertheless, first study was underpowered due to a small sample size of only 198 women and results were not adjusted using multivariable regression analysis [12]. 

Further reports described no effect of BMI on complication rates for vaginal hysterectomy or TVT [14–16]. Our study, with its larger sample size and statistical power using multivariable regression analysis, further reinforces findings of no effect of BMI on incidence of bladder injury and urinary retention. Multivariable regression analysis has become a standard statistical method for analysis of retrospective studies in obstetrics and gynaecology as it is a useful way of describing the relationship between one or more potential risk factors such as obesity and an outcome such as bladder injury [17, 18].

There are some sources of potential bias in this study. Data was collected retrospectively from the hospital database, so there is a chance some data could have been entered inconsistently. However, this means that data were entered without knowledge of a study hypothesis therefore reducing the risk of bias. There may be additional outstanding factors that could have an effect on the results that have not been included in our analysis. Lastly, these results are only from one hospital, so it is possible that other hospitals would have different findings. However, this is unlikely, as the incidence of complications appears to be consistent with other studies [9, 10, 19]. In conclusion, based on our statistical analysis, it appears that obesity is not an independent risk factor for a bladder injury or urinary retention following TVT.

## Figures and Tables

**Table 1:** Characteristic of cohort by body mass index (BMI).

Characteristics	BMI		
	<30 (*n* = 246), 72%	≥30 (*n* = 96), 28%	*P* value
Median (IQR) age	54 (45–64)	55 (44.75–64)	.7837
Concomitant procedure			
No.	182 (74%)	75 (78%)	.51
A&P repair	42 (17%)	13 (14%)	.5254
VH and repair	22 (9%)	8 (8%)	.9732
Analgesia			
GA	8 (3%)	4 (4%)	.9314
Spinal	105 (43%)	38 (40%)	.689
Sedation	133 (54%)	54 (56%)	.8073
Previous surgery			
No.	219 (89%)	88 (92%)	.599
TAH	13 (5%)	4 (4%)	.8803
VH and repair	9 (4%)	3 (3%)	.9314
Burch	5 (2%)	1 (1%)	.8659
Complications			
No.	209 (85%)	86 (90%)	.3466
Bladder damage	13 (5%)	3 (3%)	.5722
Retention of urine	24 (10%)	7 (7%)	.6145

IQR: interquartile range; A&P: anterior and posterior; GA: general anaesthesia; TAH: total abdominal hysterectomy; VH: vaginal hysterectomy.

**Table 2:** Unadjusted and adjusted odds ratios (OR) (95% confidence interval) for specific complication by body mass index (BMI).

	Unadjusted OR		Adjusted OR*	
BMI	Bladder injury	Retention	Bladder injury	Retention
<30/≥30	1.7296 (0.4818–6.2097)	1.3745 (0.5718–3.3043)	2.885 (0.603–13.8)	1.299 (0.502–3.365)
*Adjusted for age, concomitant surgery, analgesia, previous surgery				
McFadden R-Squared 0.085, and posthoc statistical power 0.996				
